# Genomic and virulence characteristics of *Staphylococcus aureus* isolates from foodborne outbreak cases

**DOI:** 10.1186/s12866-026-05310-2

**Published:** 2026-06-25

**Authors:** Li Zhou, Yixin Zhang, Qian Zhou, Lian Zheng, Shu Zhu, Jingshu Xiang, Xinxu Luo, Ying Liu, Shijun Li

**Affiliations:** 1 Guizhou Provincial Centre for Disease Control and Prevention, Guizhou, China; 2 The Second Affiliated Hospital of Chongqing Medical University, Chongqing, China; 3 School of Public Health, the Key Laboratory of Environmental Pollution Monitoring and Disease Control, Ministry of Education, Guizhou Medical University, Guizhou, China

**Keywords:** *Staphylococcus aureus*, Virulence genes, Antibiotic resistance, Mobile genetic elements, Genetic diversity

## Abstract

This study aimed to investigate the genomic characteristics, enterotoxin production, and antimicrobial resistance profiles of *Staphylococcus aureus* isolates associated with foodborne outbreaks. A total of 19 bacterial isolates were collected from foodborne outbreaks in Guizhou Province, China between 2014 and 2023. Following biochemical identification, all isolates were confirmed as *S. aureus*. Phylogenetic analysis divided the 19 strains into seven branches. Enterotoxin production was detected using standard microbiological techniques and immunoassays. Antimicrobial susceptibility was evaluated using the broth microdilution method. Whole-genome sequencing and subsequent bioinformatic analyses were conducted to characterize virulence genes, antimicrobial resistance genes, multilocus sequence typing (MLST) genotypes, and phylogenetic relationships among the isolates. This study found that all strains produced classical staphylococcal enterotoxins, with staphylococcal enterotoxin (SEA) showing the highest detection rate (63.16%). Virulence gene profiling revealed widespread presence of *hlb*, *hlgA*, *nuc*, *clfB*, *spa*, and *set* genes. All strains were resistant to penicillin, with high resistance rates for erythromycin and cefoxitin. Multidrug resistance occurred in 11 of the 19 strains, and 22 resistance genes were identified. MLST analysis showed that ST6 and ST59 were the dominant types, with ST59 methicillin-resistant *S. aureus* (MRSA) strains displaying stronger resistance and more virulence determinants. These findings provide insights into the virulence, resistance, and molecular epidemiology of *S. aureus* strains involved in foodborne outbreaks, and may provide useful information for future surveillance and risk assessment.

**Supplementary Information:**

The online version contains supplementary material available at https://doi.org/10.1186/s12866-026-05310-2.

## Introduction

Food-related health issues caused by foodborne diseases (FBD) are a major public health concern [4, 34]. FBD encompass a wide range of illnesses, including infectious diseases caused by microorganisms and toxic diseases primarily induced by chemical and toxic substances. The World Health Organization (WHO) estimated that in 2010, up to 600 million people (almost 1 in 10 individuals) suffered from FBD [20, 28]. In China, *Staphylococcus aureus* is the third leading pathogen responsible for FBD. In 2021, *S. aureus* accounted for 10.6% of bacterial food poisoning incidents, imposing a significant disease burden [22].

*S. aureus* is recognized as one of the leading causes of foodborne diseases globally and can persist in food through complex mechanisms [41]. It is estimated that *S. aureus* accounts for 20–25% of bacterial foodborne outbreaks in China [46]. In Guizhou province, *S. aureus* ranks as the second most common microbial cause of FBD outbreaks. This pathogen produces a variety of toxins and invasive enzymes, with staphylococcal enterotoxins (SEs) being the primary virulence factors (VFs) associated with food poisoning [18, 30, 33]. Multiple antigenic SE types have been identified, including staphylococcal enterotoxin A (SEA), SEB, SEC, SED, SEE, SEG, SEH, SEI and SEJ, many of which have been associated with gastrointestinal symptoms. In addition, *S. aureus* produces several other VFs, such as toxic shock syndrome toxin (TSST), exfoliative toxins, and leukocidins, which contribute to invasive infections in humans. These infections can lead to suppurative diseases, including skin and soft tissue infections, necrotizing pneumonia, sepsis, and toxic shock syndrome [1, 11, 16, 38, 39]. Furthermore, certain methicillin-resistant *S. aureus* (MRSA) lineages associated with infectious diseases harbor distinct virulence genes [54].

Studies have shown that VFs may contribute to the virulence of isolates. Notably, enterotoxin-producing strains are the primary cause of food poisoning, which is associated with the consumption of food containing sufficient levels of SEs [32]. Genes encoding SEs can be located on various genetic elements, including pathogenicity islands (*seb*, *sec*), prophages (*sea*, *see*, *sep*), transposons (*seb*), and plasmids (*sed*, *sej*, and *ser*) [6, 12, 26, 37]. The presence of VF genes on mobile genetic elements (MGEs) is particularly significant, as horizontal gene transfer (HGT) is one of the most crucial mechanisms in the food chain for the exchange of antimicrobial resistance (AMR) and virulence determinants within bacterial populations [18, 42]. Notably, AMR has become a rapidly escalating global threat to humans, animals, and the environment. The complexity of resistance mechanisms and the abundance of MGEs have significantly contributed to the rapid evolution of multidrug-resistant (MDR) strains [23, 30]. Additionally, increasing evidence indicates that the prevalence of antibiotic resistance genes (ARGs) in animals and their potential horizontal transfer to humans are increasing, posing serious public health risks [40, 53]. However, current research on the horizontal transfer of virulence and resistance genes via genetic elements in foodborne *S. aureus* isolates remains limited.

This study conducted a comprehensive analysis of toxin production, AMR, and whole-genome sequencing on *S. aureus* isolated from 19 FBD outbreaks in Guizhou from 2014 to 2023. By elucidating the pathogenic and molecular genetic characteristics of these *S. aureus* strains, this research provides useful insights into the prevention and control of *S. aureus*-induced food poisoning and contributes to a better understanding of its epidemiological characteristics.

## Materials and methods

### Sources and identification of bacterial isolates

A total of 19 bacterial isolates were collected from 19 FBD outbreaks that occurred in Guizhou Province between 2014 and 2023. The outbreaks were distributed across five cities/prefectures: Guiyang (*n* = 5), Anshun (*n* = 6), Tongren (*n* = 3), Zunyi (*n* = 2), and Qiannan (*n* = 3). Detailed case and sample information is provided in Table S1. The isolates were initially cultured on Baird-Parker agar plates (Guangdong Huankai Microbial Sci. & Tech. Co., Ltd, China) for selective isolation and on blood agar plates (Huankai Microbial, Guangzhou, China) to observe hemolytic activity. Preliminary identification was performed using the Gram-positive bacterial identification card (BioMérieux, Marcy-l’Etoile, France) via the VITEK 2-compact system. Final confirmation of *S. aureus* isolates was performed according to the National food safety standard Food microbiological examination: *Staphylococcus aureus* (GB 4789.10–2016) [15]. The steps included hemolysis observation, coagulase testing, biochemical characterization, and Matrix-Assisted Laser Desorption/Ionization – Time of Flight Mass Spectrometry (MALDI-TOF MS) (Bruker Daltonics, Bremen, Germany) identification. Confirmed *S. aureus* isolates were preserved in 20% glycerol stocks at − 80 °C for subsequent analyses.

### Detection and typing of SEs

The detection and typing of SEs were conducted using the Mini VIDAS fluorescence immunoassay analyzer (BioMérieux, Marcy-l’Etoile, France), according to the *Examination codes for biological toxin at entry-exit port, Part 2: Staphylococcal Enterotoxin B* (S/NT 1763[1]0.2—2006) [19], and National food safety standard Food microbiological examination: Staphylococcus aureus (GB 4789.10–2016) [15]. Isolates were enriched in CAMHT broth medium (Kanglang Biotechnoloty, Shanghai, China) prior to analysis. SEs were detected using the VIDAS Staphylococcal Enterotoxin Detection Kit II (SET 2, BioMérieux, Marcy-l’Etoile, France), and specific SE types were further identified with the Staphylococcal Enterotoxin Typing Kit (Meizheng Biotechnology, Beijing, China). All experiments included positive controls and negative controls consisting of sterile brain heart infusion (BHI) broth.

### Antimicrobial susceptibility testing

Antimicrobial susceptibility of the isolates was determined using the broth microdilution method, following the guidelines of the Clinical and Laboratory Standards Institute (CLSI) and Chinese *2023 Foodborne Disease Surveillance Manual*. A total of 15 antimicrobial agents were tested, including cefoxitin (CFX), oxacillin (OXA), penicillin (PEN), tetracycline (TET), erythromycin (ERY), clindamycin (CLI), gentamicin (GEN), trimethoprim-sulfamethoxazole (SXT), linezolid (LIN), daptomycin (DAP), levofloxacin (LEV), teicoplanin (TEI), rifampin (RIF), nitrofurantoin (NIT), and vancomycin (VAN). *S. aureus* ATCC 29213 was used as the quality control strain.

### Whole genome sequencing

Bacterial DNA was extracted following the instructions of the TaKaRa MiniBEST Bacteria Genomic DNA Extraction Kit (v.3.0; TaKaRa, Shiga, Japan). The genomic DNA of the strains was sent to Sangon Biotech Co.,Ltd for second-generation sequencing using the DNBSEQ-T7 platform (MGI Tech Co., Ltd, Beijing, China). The sequencing depth was 200 ×. FastQC was used for sequencing quality control, and Bionumerics database software (v. 7.6; Applied Maths BVBA, Belgium) was used for sequence assembly.

### Bioinformatics analysis

The virulence genes carried by the isolates were screened using the virulence gene database (http://www.mgc.ac.cn/VFs/search_VFs.html) [29] and the VirulenceFinder database (https://cge.cbs.dtu.dk/services/VirulenceFinder/) [9, 14]. The resistance genes carried by the isolates were identified using the Comprehensive Antibiotic Resistance Database (CARD: http://card.mcmaster.ca/) [2]. The presence of MGEs was predicted using the VRprofile2 database (https://tool2-mml.sjtu.edu.cn/VRprofile/VRprofile.php) [45]. Multi-locus sequence typing (MLST) of the isolates was performed using the *S. aureus* MLST database (https://pubmlst.org/organisms/staphylococcus-aureus), which assigns sequence types (STs) based on seven housekeeping genes (*arcC*, *aroE*, *glpF*, *gmk*, *pta*, *tpi*, *yqiL*) [25]. The *spa* types of the isolates were determined using spaTyper 1.0 of the Center for genomic epidemiology (https://cge.food.dtu.dk/services/spaTyper/) [7]. SCCmec typing was conducted using the SCCmecFinder platform (https://cge.food.dtu.dk/services/SCCmecFinder/) [36]. Core genome sequences of the isolates were compared using the Bacterial and Viral Bioinformatics Resource Center (BV-BRC: https://www.bv-brc.org/). A phylogenetic tree was constructed based on core genome alignments and further visualized and refined using tvBOT (https://www.chiplot.online/tvbot.html) [48] and Chiplot (http://www.evolgenius.info/) [43]. Single nucleotide polymorphism (SNP) analysis of the *S. aureus* isolates was conducted using Snippy v4.6.0, with the complete genome of the reference strain *S. aureus* NCTC 8325. A phylogenetic tree was then constructed based on SNP data using IQ-TREE.

### Statistical analysis

Pearson correlation analysis was performed, with the correlation coefficient represented by r (|r|< 0.4 indicating weak correlation, 0.4 ≤|r|≤ 0.7 indicating moderate correlation, and |r|> 0.7 indicating strong correlation). A *P*-value less than 0.05 was considered statistically significant (Fisher’s exact test was used when the expected frequency was less than 5). The correlation heatmap was generated using Origin 2021. The Sankey diagram and Manhattan plot were created using the sankeyNetwork function and the ggplot2 package in R (v.4.4.1), respectively.

## Results

### Whole-genome sequencing reveals the phylogenetic diversity of outbreak-associated *S. aureus*

**Fig. 1:**
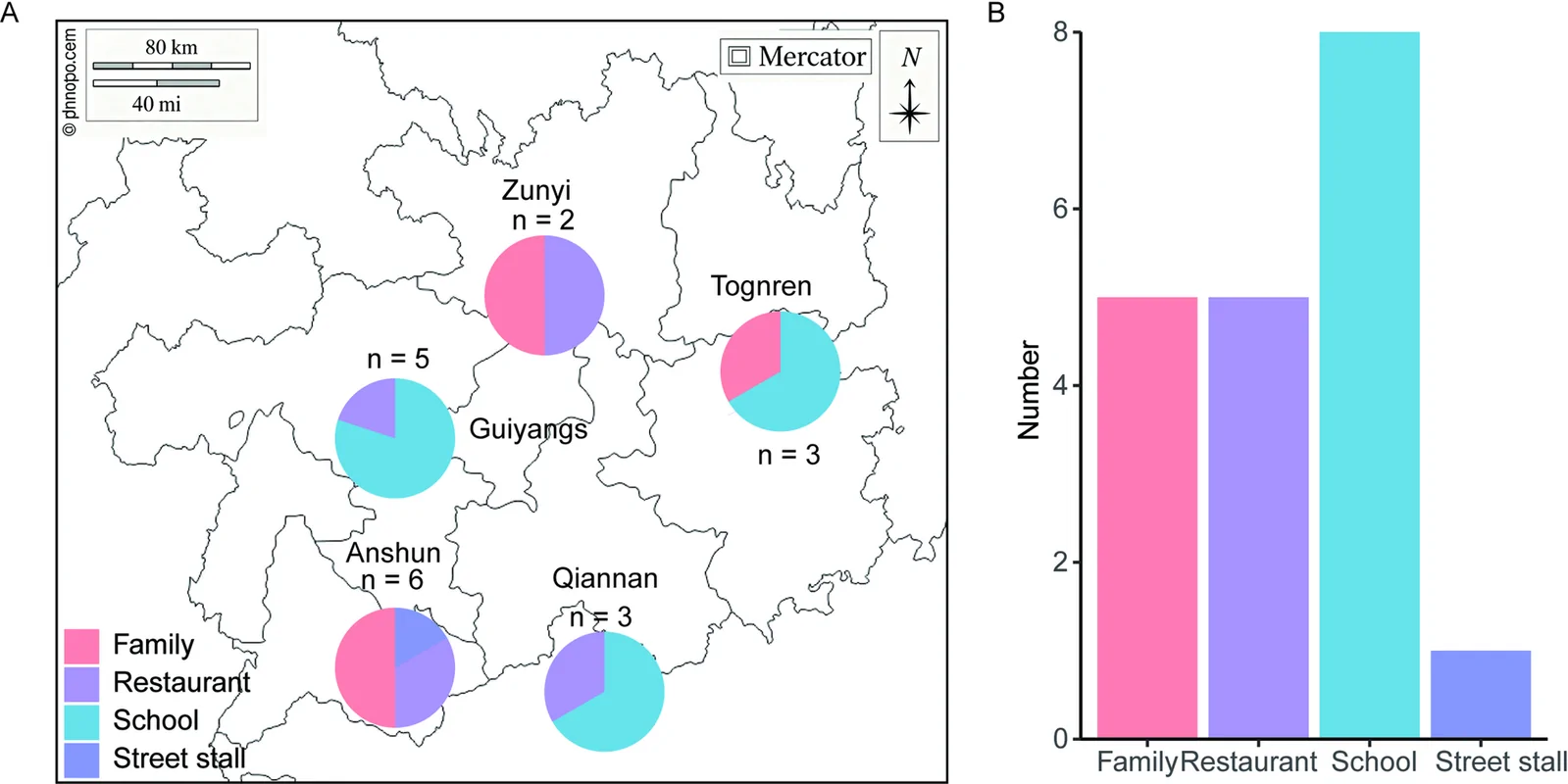
Spatial distribution characteristics of *Staphylococcus aureus* isolated from foodborne outbreaks in Guizhou. **A** The map shows the geographical locations of the 19 *S. aureus* strains isolated from foodborne outbreak incidents. **B** The bar chart illustrates the number of *S. aureus* strains isolated from different types of locations

Between 2014 and 2023, a total of 19 isolates were collected in Guizhou Province, China, and confirmed as *S. aureus* using the VITEK 2-compact system and mass spectrometry analysis. These strains were involved in outbreaks at four types of locations: households (*n* = 5), restaurants (*n* = 5), schools (*n* = 8), and street food stalls (*n* = 1) (Fig. 1).

**Fig. 2:**
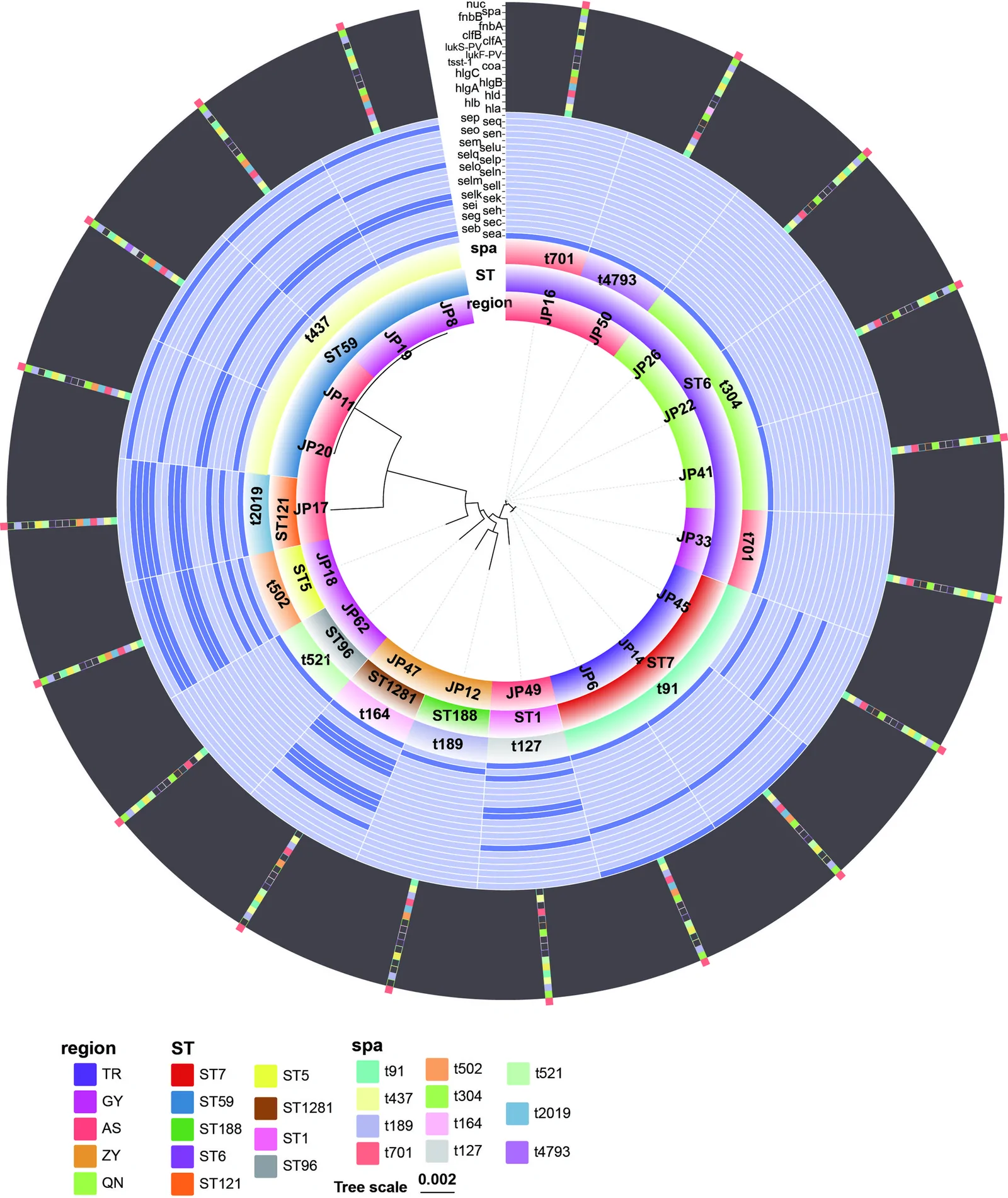
Phylogenetic tree of the 19 *S. aureus* strains

A total of 19 *S. aureus* genomes were successfully assembled. For most genomes, the number of contigs ranged from 9 to 83, whereas isolate JP14 was an outlier with 1,804 contigs. The total assembly lengths varied between 2.77 and 5.27 Mb, and the N50 values ranged from 2.3 kb to 730 kb, reflecting variable assembly continuity. The longest contigs spanned 110 kb to 833 kb, and GC content was generally consistent, ranging from 32.55% to 32.8%, except for two genomes with higher GC content (JP11: 51.94%, JP8: 35.1%). Overall, the genome assemblies of the 19 *S. aureus* isolates showed sufficient quality for downstream molecular typing and comparative genomics analyses (Table S2). Phylogenetic analysis indicated that the 19 strains were divided into seven branches. Strains within the same branch showed high homology. Specifically, JP16, JP50, JP26, JP33, JP22, and JP41 formed one branch; JP6, JP14, and JP45 formed another branch; JP47 and JP12 formed a third branch; JP8, JP19, JP11, JP20, and JP17 formed a fourth branch; and JP18, JP62, and JP49 each formed a separate branch (Fig. 2).

### Molecular typing reveals diverse sequence and *spa* types with a dominant MRSA lineage among *S. aureus* isolates

A total of nine ST types were identified from the 19 strains, including: ST6 (six strains, 31.6%), ST59 (four strains, 21.05%), ST7 (three strains, 15.79%), and ST1, ST5, ST96, ST121, ST188, and ST128 each with one strain (5.26%). In contrast, 11 *spa* types were identified, including: t437 (four strains, 21.1%), t91 (three strains, 15.79%), t304 (three strains, 15.79%), t701 (two strains, 10.53%), t189, t2019, t502, t164, t127, t4793, and t521 each with one strain. The three strains of t91 and t304 were from Tongren and Qiannan, respectively, while the strains of t437 and t701 were from Guiyang and Anshun. The strains with *spa* types t304, t701, and t4793 were all ST6, while other *spa* types corresponded to a specific ST type. All four t437 strains were ST59, as shown in Table S3. Three MRSA strains were detected and subjected to SCCmec typing. The results showed that all the three MRSA isolates shared identical molecular profiles: SCCmec IVa (2B), ST59, and *spa* type t437.

### Virulence profiling reveals extensive pathogenic diversity in *S. aureus* isolates

The detected enterotoxin types of the 19 strains were SEA, SEB, and SEC. The detection rates of the three enterotoxins were as follows: SEA, 63.16% (12/19); SEB, 31.58% (6/19); and SEC, 5.26% (1/19). The enterotoxin detection results for the 19 strains from five cities (prefectures) are shown in table S4. According to gene annotation results from the VFDB and VirulenceFinder virulence factor databases, the 19 *S. aureus* strains contained varying numbers of VFs, ranging from 10 to 27. These included genes encoding superantigen exotoxins, cytotoxins, extracellular enzymes, and adhesion-related pathogenic factors. The detection rates of *hlb*, *hlgA*, *nuc*, *clfB*, *spa*, and set were 100%, while *fnbA* and *fnbB* were detected in 73.68% and 42.11% of the strains, respectively. One strain (JP50) tested positive for tsst-1, and one strain (JP11) tested positive for *lukF-PV* and *lukS-PV*. Details are shown in table S5.

**Fig. 3:**
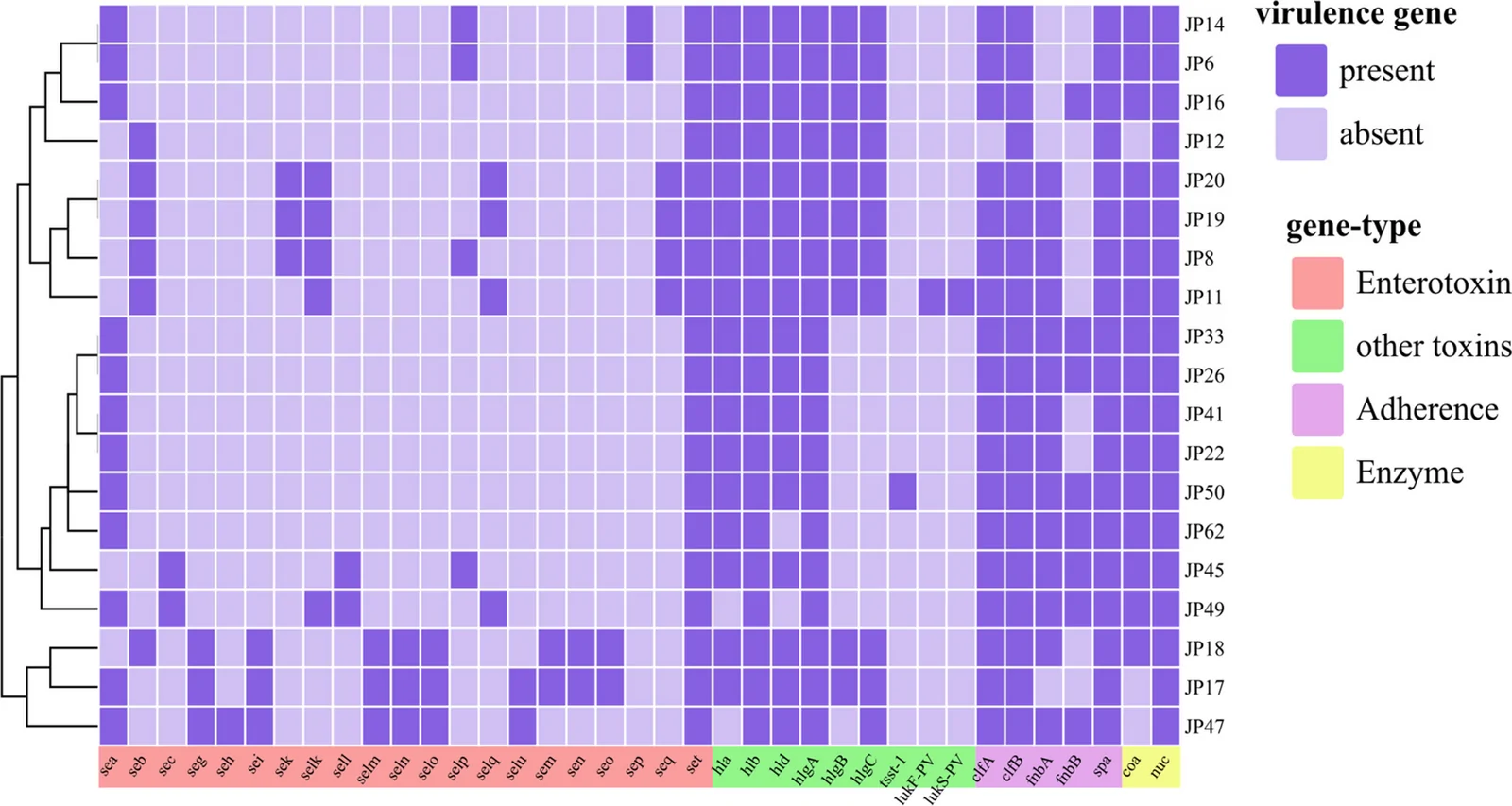
Heatmap of the carriage of virulence factor in 19 *S. aureus* strains

The 19 strains carried between one and ten enterotoxin genes. The detected enterotoxin genes included classical enterotoxin genes (*sea-sec*), newly identified enterotoxin genes (*seg*, *seh*, *sei*, *sek*, *SEM-seq*), and enterotoxin-like genes (*selk-selq*, *sel*), totaling 12 enterotoxin genes and 8 enterotoxin-like genes. Among them, *sea* had the highest detection rate at 63.16% (12/19), followed by *seb* detected in six strains (31.58%) and *selk* detected in five strains (26.31%). The presence of *sea* and *seb* was consistent with the expression of SEA and SEB. One strain carried both *sea* and *sec*, but only SEA was detected. The 19 strains exhibited 11 different enterotoxin gene profiles: *sea* was present in 36.57% (7/19) of the strains; *sea-selp-sep* and *seb-sek-selk-selq-seq* were each present in 10.53% (2/19); and *seb*, *sec-sell-selp*, *seb-selk-selq-seq*, *seb-sek-selk-selp-seq*, *sea-sec-seh-selk-sell-selq*, *sea-seg-sei-selm-seln-selo-sulu*, *seb-seg-sei-selm-seln-selo-SEM-sen-seo*, and *sea-seg-sei-selm-seln-selo-sulu-SEM-sen-seo* were each present in 5.26% (1/19) of the strains. No strain was found to carry both *sea* and *seb* simultaneously. All six *seb*-carrying strains tested negative for *fnbB* (Fig. 3).

### Resistance profiling identifies multidrug-resistant and genetically heterogeneous *S. aureus* isolates

The antimicrobial susceptibility of the 19 *S. aureus* strains was assessed to determine their resistance profiles and identify MDR strains. All the strains were β-lactamase positive, exhibiting varying degrees of resistance to PEN, OXA, CFX, CLI, TET, GEN, ERY, DAP, and NIT. The resistance rates were as follows: 100.00% (19/19) for PEN, 26.32% (5/19) for OXA, 36.84% (7/19) for CFX, 31.58% (6/19) for CLI, 21.05% (4/19) for TET, 5.26% (1/19) for GEN, 47.37% (9/19) for ERY, 15.79% (3/19) for DAP, and 5.26% (1/19) for NIT. None of the strains were resistant to VAN, TEI, RIF, LEV, SXT, or LIN.

A total of 11 antimicrobial resistance profiles were identified (Table 1). MDR strains, defined as resistant to three or more classes of antibiotics, accounted for 63.16% (12/19). The remaining isolates exhibited resistance to only one or two antibiotics and were thus classified as non-MDR.

**Table 1:** Antimicrobial resistance profiles

Profile	Number of strains	Percentage (%)
PEN	7	36.84
CLI + ERY + PEN	2	10.53
TET + ERY + PEN	2	10.53
OXA + PEN	1	5.26
DAP + PEN + CFX	1	5.26
TET + DAP + PEN	1	5.26
CLI + ERY + PEN + CFX	1	5.26
OXA + CLI + ERY + PEN + CFX	1	5.26
OXA + ERY + PEN + NIT + CFX	1	5.26
OXA + CLI + GEN + ERY + PEN + CFX	1	5.26
OXA + CLI + TET + ERY + PEN + CFX	1	5.26

*PEN* penicillin, *CLI* clindamycin, *ERY* erythromycin, *TET* tetracycline, *OXA* oxacillin, *DAP* daptomycin, *CFX* cefoxitin, *GEN* gentamicin, *NIT* nitrofurantoin

A total of 22 ARGs across eight categories were identified, including: β-lactam resistance genes *mecA* (26.32%) and *blaZ* (84.21%), macrolide resistance genes *ermB* (31.58%) and *ermC* (26.32%), TET resistance genes *tet(K)* (26.32%), *tetR* (5.26%), *tet(38)* (84.21%), and *tet(45)* (5.26%), quinolone resistance genes *norA* (47.37%) and *norC* (36.84%), aminoglycoside resistance genes *ant(4')-Ib* (52.63%), *ant(6)-Ia* (21.05%), *aph(3')-III* (21.05%), *aph(3')-IIIa* (10.53%), *aadD* (10.53%) and *aad(6)* (15.79%), lincosamide resistance genes *lnuA* (15.79%) and *lnuG* (5.26%), fosfomycin resistance genes *fosB* (47.37%) and *fosK* (5.26%), and trimethoprim resistance genes *dfrC* (47.37%) and *dfrG* (10.53%). Combining the resistance phenotype results, JP8 and JP19 from Guiyang, and JP11 from Anshun were identified as MRSA. These three strains were all MDR, with resistance profiles of OXA + NIT + ERY + PEN + CFX, OXA + CLI + TET + ERY + PEN + CFX, and OXA + CLI + ERY + PEN + CFX, respectively.

**Fig. 4:**
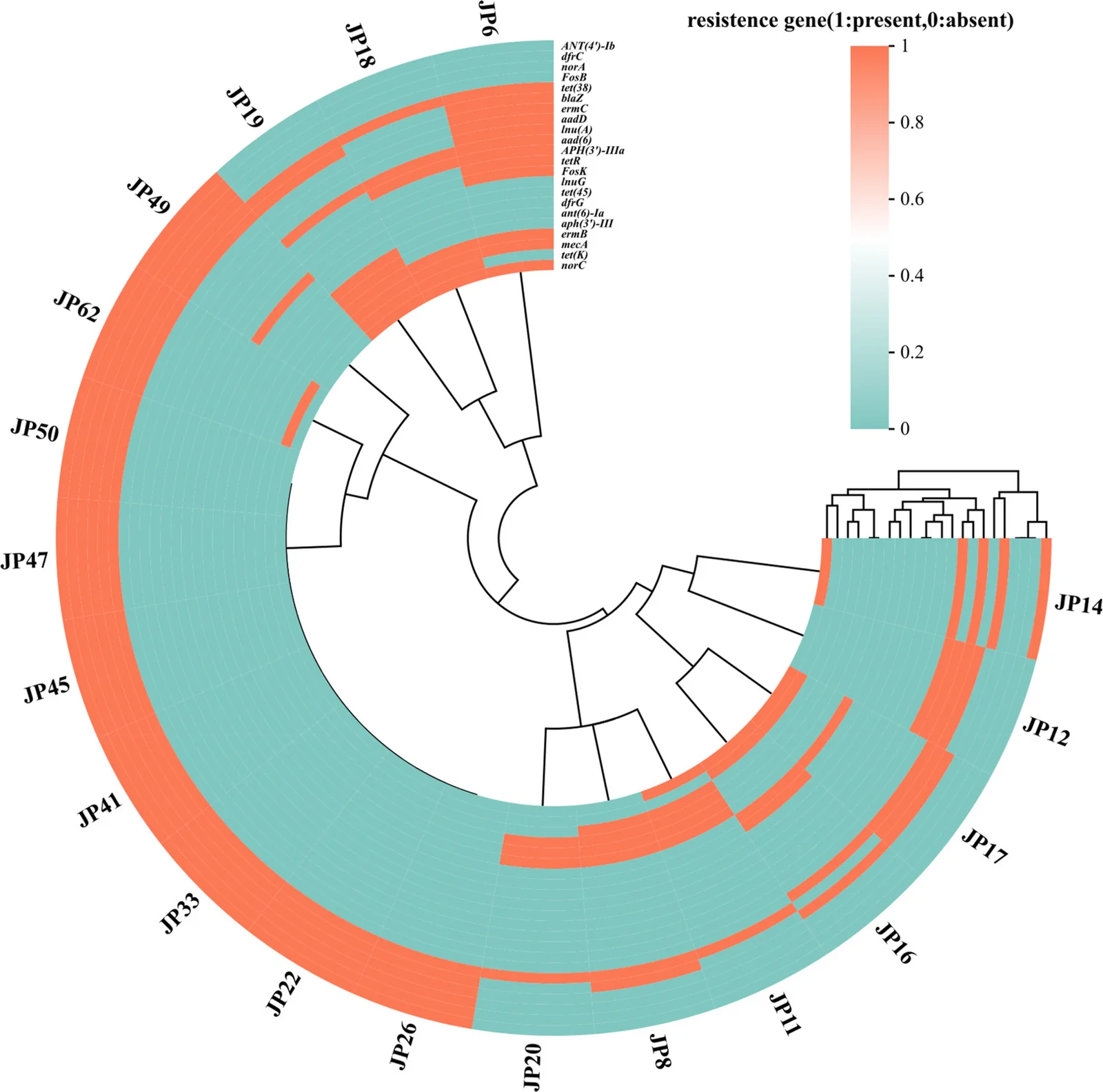
Antibiotic resistance gene profiles of the 19 *S. aureus* strains

The ARG profiles of the 19 strains were investigated, resulting in 13 distinct profiles. Seven strains shared an identical resistance gene profile, including JP33 from Guiyang, JP20 and JP50 from Anshun, JP45 from Tongren, and JP22, JP26, and JP41 from Qiannan (Fig. 4).

### MGEs mediate virulence and antibiotic resistance in *S. aureus* isolates

#### Detection of MGEs reveals regional variation among strains

**Fig. 5:**
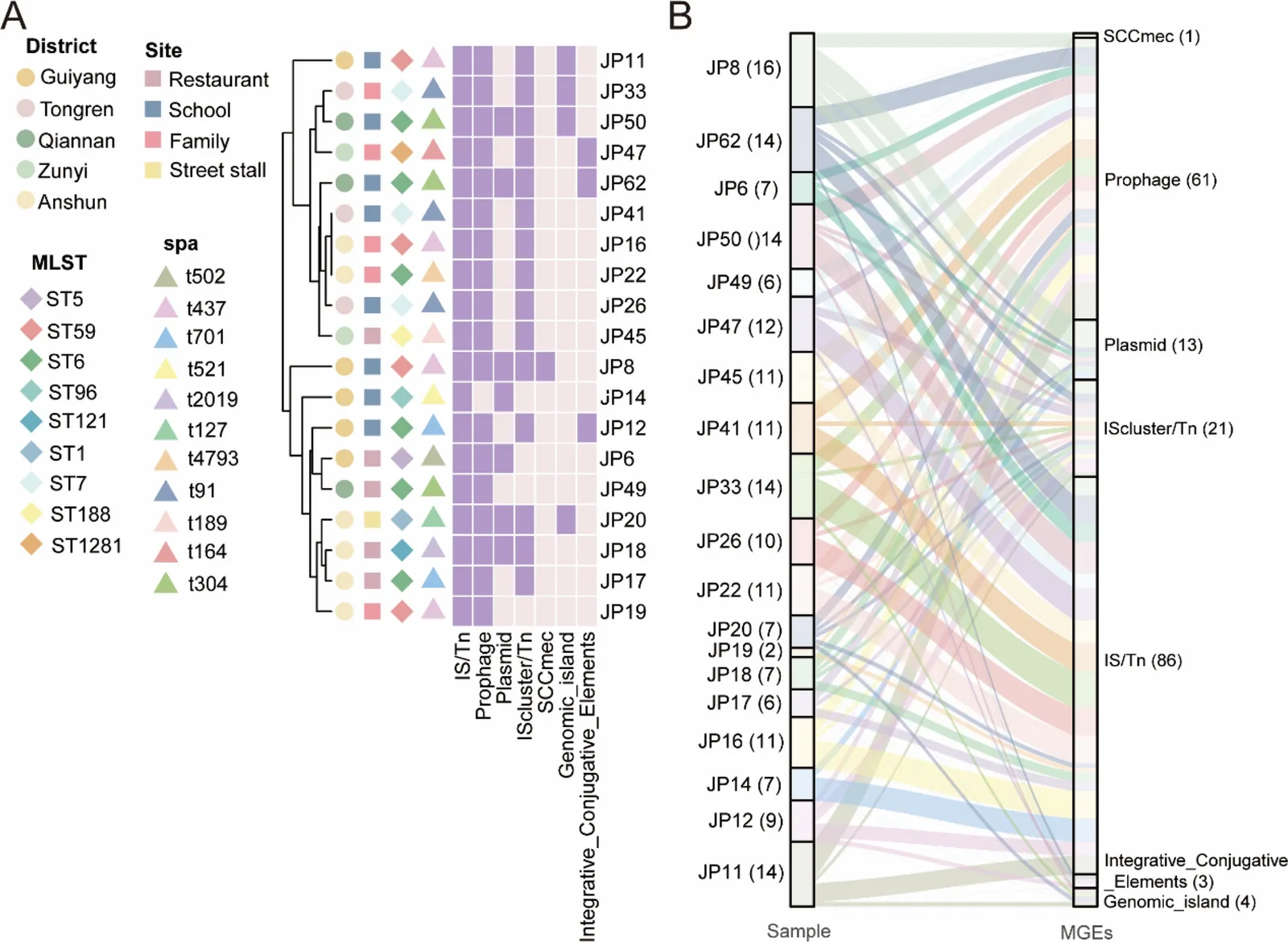
Detection results of mobile genetic elements (MGEs). **A** MGE profile of 19 *S. aureus* strains. **B** Association between each strain and MGEs

Based on the VRprofile2 database, prediction of MGEs in the 19 *S. aureus* strains identified seven types of MGEs: insertion sequences/transposons (IS/Tn), prophages, plasmids, insertion sequence clusters/transposons (ISCluster/Tn), SCCmec (methicillin resistance gene island), integrative conjugative elements, and genomic islands. Among these, IS/Tn was detected in all 19 strains (100%), prophage was not detected in JP14 (94.74%), and SCCmec was only detected in JP8 (5.26%) (Fig. 5A). The association between the strains and specific MGEs showed that the types and quantities of MGEs carried by the strains vary greatly across different regions. For example, JP8 isolated in Guiyang was associated with a larger number and variety of MGEs, while JP19 isolated in Anshun was associated with fewer types and quantities of MGEs (Fig. 5B).

#### Association of MGEs with virulence and resistance genes

**Fig. 6:**
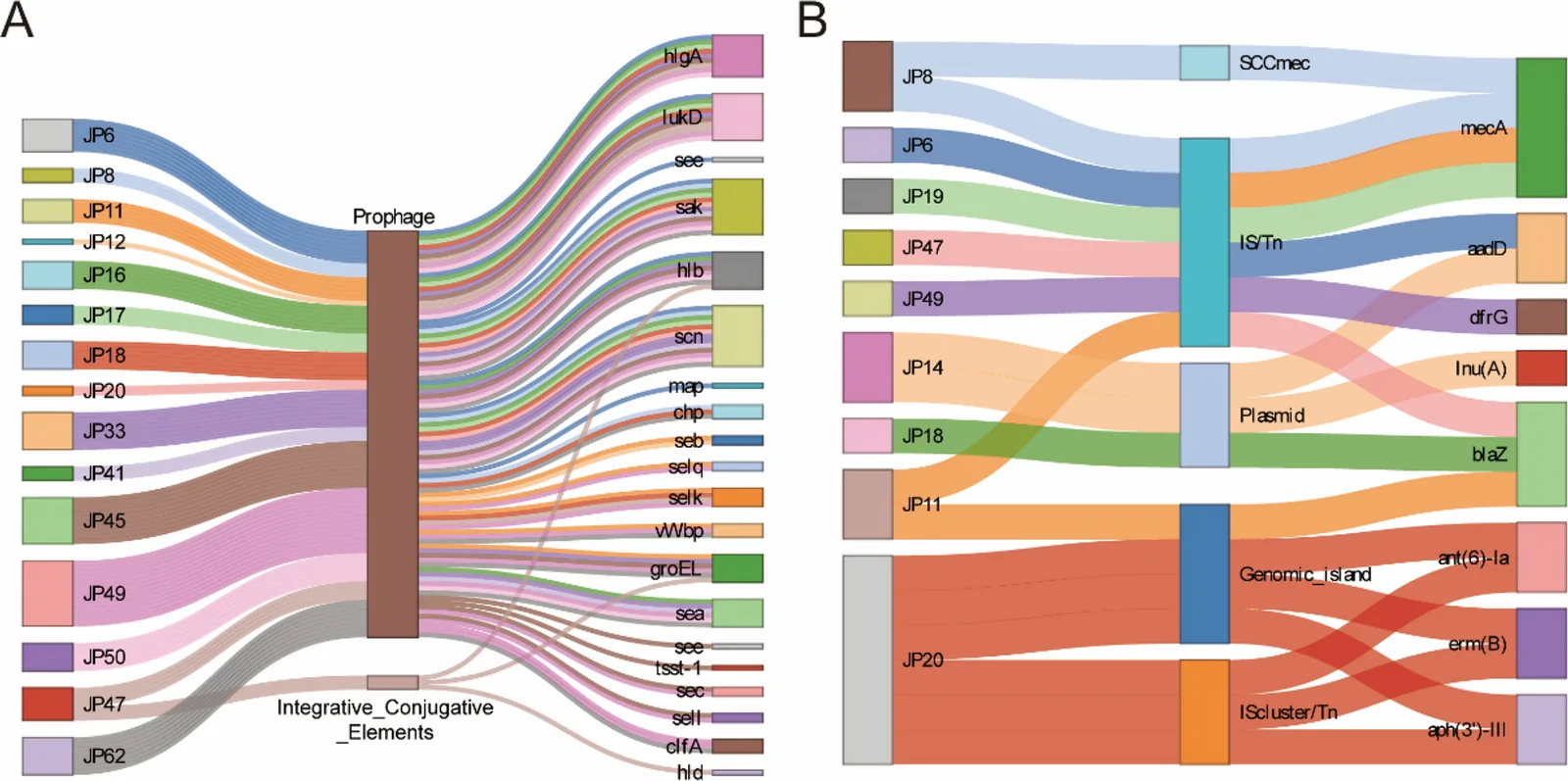
Association between MGEs and VFs (**A**) and ARGs (**B**)

To understand the distribution of the predicted MGEs in different strains, the association of these MGEs with VFs and ARGs was analyzed using the VRprofile2 database. According to the results, MGEs of all 15 strains carried virulence genes, with 3.33% of VFs being carried by integrative conjugative elements, while the remaining VFs carried by prophage. Notably, the prophage in JP49 carried a greater number of VFs, indicating that prophage was a key carrier of VFs (such as sak, sen, lukD) (Fig. 6A). In the association between MGEs and ARGs, MGEs of nine strains carried resistance genes. IS/Tn was an important carrier for the spread of resistance genes, associated with multiple resistance genes (such as *aadD*, *dfrG*). Genomic islands were significantly associated with ARGs (such as *erm(B)*, *aph(3’)-III*). The distribution of these resistance genes suggested that MGEs may mediate the spread of MDR through integration and transfer mechanisms (Fig. 6B).

#### ST types are associated with virulence and antibiotic resistance in* S. aureus*

**Fig. 7:**
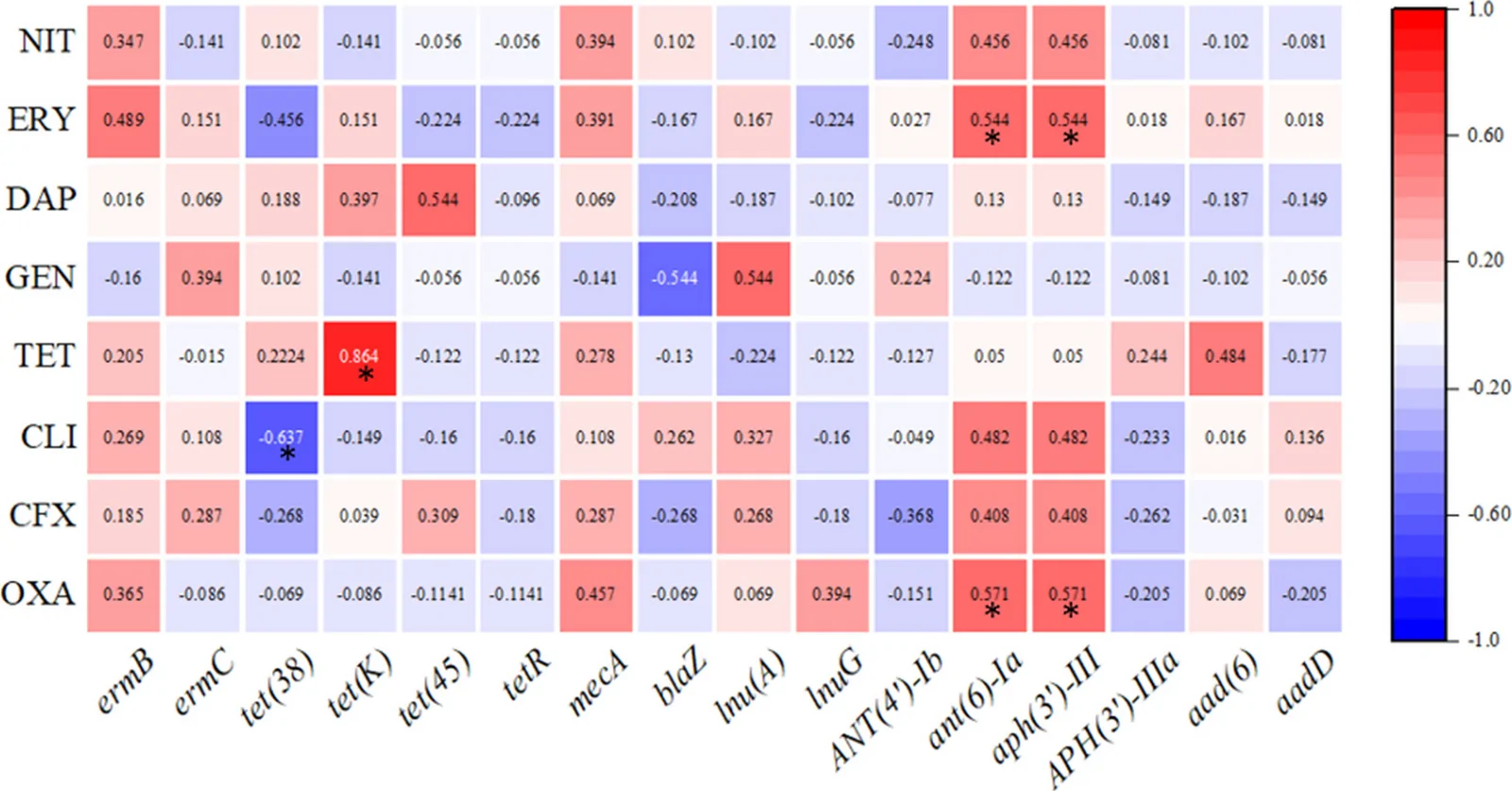
Heatmap showing the correlation between antibiotic resistance phenotypes and resistance genes

Correlation analysis between the antibiotic resistance phenotypes and ARGs showed that strains resistant to OXA and ERY were positively correlated with the resistance genes *ant(6)-Ia* and *aph(3')-III*. Those resistant to TET were positively correlated with the resistance gene *tet(K)*, while strains resistant to CLI were negatively correlated with the resistance gene *tet(38)*, with statistical significance (*P* < 0.05) (See Fig. 7).

The predominant molecular types of the 19 *S. aureus* strains, based on MLST and *spa*-typing, were ST6 and ST59 (t437 type). Correlation analysis of the predominant strains and virulence genes revealed the following results: ST6 was correlated with the enterotoxin gene *sea* (*P* < 0.001), positively correlated with the virulence gene *hld* (0.797, *P* < 0.05), and negatively correlated with the virulence gene *hlgC* (−0.567, *P* < 0.05). ST59 was associated with the enterotoxin gene profile *seb-selk-seq*, with statistically significant differences (*P* < 0.05). Analysis of the correlation between ST6, ST59 (t437 type), and resistance genes revealed that ST59 was positively correlated with the resistance genes *ermB*, *mecA*, *ant(6)-Ia*, and *aph(3')-III* (0.76, 0.571, 1.00, 1.00, respectively), and negatively correlated with the resistance gene *ant(4')-Ib* (−0.544), with statistically significant differences (*P* < 0.05). No specific pattern was observed between ST6 and the resistance genes.

### SNP analysis reveals genomic variation and regional clustering among *S. aureus* isolates

**Fig. 8:**
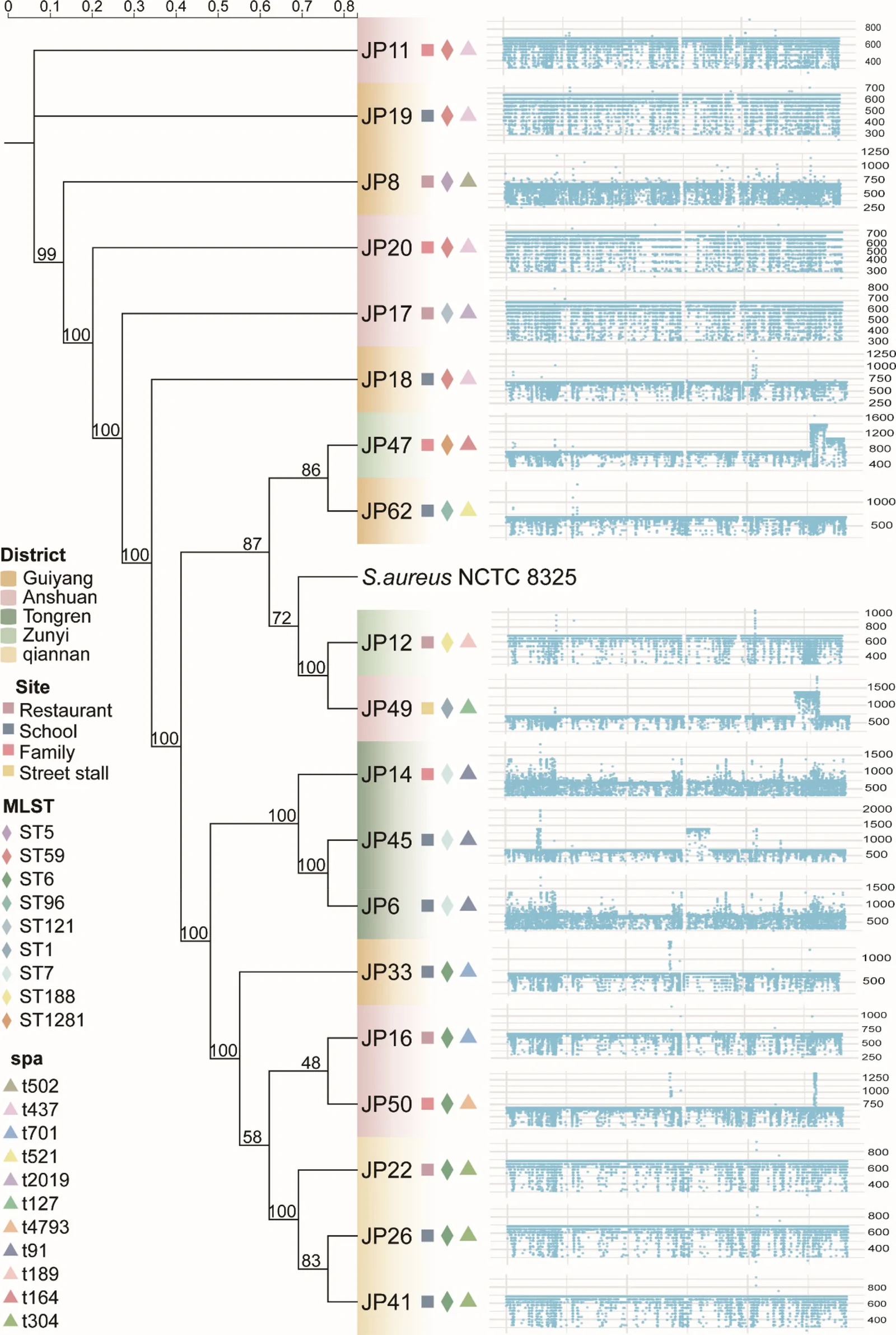
SNP Analysis. Left: Phylogenetic tree based on SNP analysis. Right: Corresponding Manhattan plot of the strains, where each point represents a variation site. The x-axis represents the length of the gene, and the y-axis represents the quality score of the SNP

In this study, SNP analysis was performed to further investigate the relationships between strains and their genomic characteristics from the perspective of genomic variation and phylogenetics. According to the phylogenetic tree based on SNP analysis, strains from different geographical regions and isolation environments exhibit clustering on the tree. For example, strains such as JP33, JP16, JP50, and JP26, primarily sourced from Zunyi and Qiannan, showed higher genetic diversity in this branch, which may reflect regional transmission and environmental adaptation. In the Manhattan plot for each strain, strains like JP11 and JP19 presented a more even SNP distribution, with higher vertical scores, indicating that the SNP sites of these strains were more reliable, possibly due to their conserved genetic backgrounds. In contrast, strains like JP50 and JP62 exhibited more dispersed SNP distributions, with prominent peaks in certain regions, which may suggest that these strains have undergone genomic rearrangements or the insertion of foreign genes. Comparison with the reference strain NCTC 8325 revealed different degrees of variation in the genomes of these strains, with these prominent variation regions potentially being important markers of HGT or genomic adaptation and evolution (Fig. 8).

## Discussion

*S. aureus* is a type of bacteria that can cause severe infections in humans, and it has been classified by the WHO as one of the bacteria that poses a significant threat to human health [17]. In this study, among the 19 strains of *S. aureus*, classic enterotoxins SEA, SEB, and SEC were detected, with SEA being the most prevalent at 63.16%. This finding is consistent with reports of the most common enterotoxins involved in global staphylococcal food poisoning, which are SEA, SEB, and SEC [13]. Virulence gene analysis showed that, aside from 8 strains carrying only one enterotoxin gene, the remaining 11 strains carried three or more (similar) enterotoxin genes, with the highest strain carrying 10 types of (similar) enterotoxin genes. In addition, most strains also tested positive for various toxin genes such as hemolysin, extracellular enzymes, and adhesion-related genes. This suggests that these *S. aureus* strains, while causing gastrointestinal symptoms in FBD, may also lead to skin and soft tissue infections, endocarditis, and even bacteremia, sepsis, and other systemic infections in the infected individuals.

The antibiotic susceptibility testing results demonstrated that all 19 strains of *S. aureus* were resistant to PEN, and the resistance rates to ERY, CFX, CLI, TET, and other antibiotics were higher than those of isolates from the food industry in China [24, 31]. The resistance rates to PEN and CFX were even higher than those of clinical strains monitored in China. However, in contrast to the 10–30% resistance rate to LEV in clinical strains, all 19 strains in this study were sensitive to LEV, suggesting that LEV could be a more ideal therapeutic option for foodborne *S. aureus* poisoning in this region. ARG detection revealed that no resistance genes for DAP and NIT were found. However, resistance genes for other antibiotics were detected.

Correlation analysis between the resistance phenotypes and resistance gene profiles showed no significant correlation between the two, which is consistent with findings from Xu et al. [49]. The lack of complete correlation between the resistance phenotype and the presence of resistance genes may suggest that the existence of certain resistance genes does not necessarily indicate antibiotic resistance. One potential explanation for this inconsistency could be the lack of expression of some resistance genes, which may be influenced by various genetic and environmental conditions [3].

Aminoglycoside antibiotics play a crucial role in the treatment of staphylococcal infections. In this study, four aminoglycoside resistance genes were detected, and strains carrying the *ant(6)-Ia* and *aph(3')-III* genes were more likely to exhibit resistance to ERY and OXA. This could be because pathogenic bacteria acquire aminoglycoside-modifying enzyme resistance genes through HGT [5, 8], which, together with other resistance genes, can be transferred to plasmids, integrons, and transposons, leading to MDR. Various SE/SEl gene types from *S. aureus* have been reported, and these gene types may play a potential role in the combinations of SE/SEl genes, as these genes exist in single or multiple copies on MGEs [5, 44]. Almost all SE/SEl genes are known to reside on MGEs, such as plasmids, prophages, pathogenic islands (SaPIs), and genomic islands, and are often located adjacent to staphylococcal cassette chromosome elements [5, 21].

MLST typing analysis revealed that ST6 was the dominant type among foodborne *S. aureus* strains in this region. Previous studies have reported that ST6 strains are commonly associated with staphylococcal food poisoning outbreaks in China and frequently harbor the *sea* enterotoxin gene [50, 51]. ST59 is recognized as one of the predominant epidemic clones of community-associated MRSA in China. This lineage has been frequently identified in clinical isolates associated with skin and soft tissue infections, pneumonia, and invasive infections [27, 52]. In recent years, ST59-MRSA has shown an increasing prevalence in China and has gradually replaced traditional hospital-associated MRSA clones, such as ST239, in some regions [10]. The detection of ST59-SCCmec IVa-t437 MRSA strains in food samples in the present study suggests a potential overlap between foodborne and clinically relevant *S. aureus* lineages, highlighting the possible public health risk of transmission through the food chain.

In this study, three MRSA strains were identified as ST59, suggesting that ST59 MRSA has begun to spread widely among foodborne *S. aureus* strains in this region and should be given high attention. Phylogenetic analysis based on whole-genome sequencing and SNP analysis showed that strains of the same ST type clustered together in the same branch of the evolutionary tree, though they were isolated from different regions. Among the 4 ST59 MRSA strains identified in this study, they were isolated from two different urban areas, suggesting that strains from different regions may share the same genetic background. This also implies that ST59 MRSA strains may spread through trade, occupational contact, and livestock transportation. Similar transmission patterns have been reported for ST398 MRSA, which has been introduced from endemic regions into previously unaffected areas [35, 47].

## Conclusion

*S. aureus* strains isolated from foodborne events in Guizhou carried a variety of enterotoxins, enterotoxin-like toxins, TSST, leukocidin, hemolysin, and other toxin-related genes, as well as multiple ARGs and mobile genetic elements. The results indicate that these strains exhibited MDR, with particularly high resistance to several antibiotics. However, a weak correlation was observed between antimicrobial resistance phenotypes and genotypes. Meanwhile, mobile genetic elements were found to carry both VFs and resistance genes, suggesting their potential role in facilitating HGT, which warrants further investigation. Molecular typing analysis revealed substantial genetic diversity, with strains from different sources exhibiting close genetic relationships, suggesting possible cross-regional dissemination and genetic exchange in Guizhou. This study provides technical support for establishing a molecular database of *S. aureus* in Guizhou and may support future monitoring and risk assessment of *S. aureus*-associated foodborne diseases.

## Supplementary Information

Supplementary Material 1.

## Data Availability

The datasets generated and/or analyzed during the current study are available in the National Genomic Data Center (https://ngdc.cncb.ac.cn/?lang=zh), under BioProject accession number PRJCA059004.
