# Supplementary material for: Genomic and virulence characteristics of Staphylococcus aureus isolates from foodborne outbreak cases
Source: BMC Microbiol. 2026 Jun 25;26:792. doi: 10.1186/s12866-026-05310-2 (PMC13555856; doi:10.1186/s12866-026-05310-2)
Supplement: Supplementary file 1 [file 12866_2026_5310_MOESM1_ESM.docx]

**Table S1**. Sample strain sources

| **Region** | **Strain ID** | **Year** | **Contaminated Food** | **Location** |
| --- | --- | --- | --- | --- |
| Yunyan District, Guiyang | JP8 | 2014 | Various/Mixed Foods | Restaurant |
| Yunyan District, Guiyang | JP18 | 2015 | Fruits and Fruit Products | School |
| Huaxi District, Guiyang | JP19 | 2020 | Seafood | School |
| Huaxi District, Guiyang | JP33 | 2020 | Mixed Foods | School |
| Nanming District, Guiyang | JP62 | 2022 | Meat and Meat Products | School |
| Guanling County, Anshun | JP11 | 2014 | Bean Products | Home |
| Xixiu District, Anshun | JP16 | 2015 | Cereal Products | Restaurant |
| Xixiu District, Anshun | JP17 | 2015 | Meat and Meat Products | Restaurant |
| Xixiu District, Anshun | JP20 | 2015 | Meat and Meat Products | Home |
| Ziyun County, Anshun | JP49 | 2018 | Cereal Products | Street Stall |
| Ziyun County, Anshun | JP50 | 2023 | Seafood | Home |
| Jiangkou County, Tongren | JP6 | 2017 | Cereal Products | School |
| Yuping County, Tongren | JP14 | 2018 | Mixed Foods | Home |
| Bijiang District, Tongren | JP45 | 2019 | Cereal Products | School |
| Zunyi County, Zunyi | JP12 | 2016 | Mixed Foods | Restaurant |
| Honghuagang District, Zunyi | JP47 | 2023 | Frozen Beverages | Home |
| Weng'an County, Qiannan | JP22 | 2017 | Meat and Meat Products | Restaurant |
| Weng'an County, Qiannan | JP26 | 2020 | Mixed Foods | School |
| Huishui County, Qiannan | JP41 | 2020 | Baked Goods | School |

**Table S2**. Assembly statistics of 19 *S. aureus* genomes

| Strains | contigs | Total Length (bp) | N50 Length (bp) | Max Length (bp) | Sequence GC (%) |
| --- | --- | --- | --- | --- | --- |
| JP22 | 21 | 2808829 | 263471 | 479146 | 32.73 |
| JP26 | 23 | 2808937 | 263522 | 479146 | 32.73 |
| JP33 | 15 | 2809037 | 388325 | 850898 | 32.74 |
| JP41 | 21 | 2809453 | 227033 | 699240 | 32.73 |
| JP45 | 13 | 2964307 | 319837 | 812367 | 32.68 |
| JP47 | 15 | 3023787 | 298818 | 651560 | 32.71 |
| JP49 | 9 | 2977346 | 730165 | 776985 | 32.59 |
| JP50 | 24 | 2807839 | 263248 | 398445 | 32.75 |
| JP62 | 23 | 2800966 | 408877 | 639660 | 32.68 |
| JP6 | 23 | 2741160 | 197194 | 706983 | 32.68 |
| JP8 | 578 | 5268480 | 20539 | 110689 | 51.94 |
| JP11 | 83 | 5674252 | 203200 | 833182 | 35.1 |
| JP12 | 17 | 2756907 | 498599 | 562111 | 32.64 |
| JP14 | 1804 | 3015297 | 2290 | 34364 | 32.55 |
| JP16 | 20 | 2738548 | 371085 | 720560 | 32.67 |
| JP17 | 47 | 2743360 | 125633 | 385059 | 32.69 |
| JP18 | 44 | 2788481 | 258834 | 421737 | 32.71 |
| JP19 | 48 | 2758125 | 138103 | 452833 | 32.73 |
| JP20 | 41 | 2774342 | 149239 | 452725 | 32.8 |

**Table S3**. *spa* typing results and corresponding ST types

| MLST type | spa type (n) | Strain distribution |
| --- | --- | --- |
| ST1 | t127 (1) | JP49 |
| ST121 | t2019 (1) | JP17 |
| ST1281 | t164 (1) | JP47 |
| ST188 | t189 (1) | JP12 |
| ST5 | t502 (1) | JP18 |
| ST59 | t437 (4) | JP11, JP20, JP8, JP19 |
| ST6 | t304 (3) | JP22, JP26, JP41 |
|  | t701 (2) | JP16, JP33 |
|  | t4793 (1) | JP50 |
| ST7 | t91 (3) | JP6, JP14, JP45 |
| ST96 | t521 (1) | JP62 |

**Table S4**. Expression of classical enterotoxins in 19 *S. aureus* food poisoning strains

| Enterotoxin | Number of Strains (n) | Strain source (n) | | | | |
| --- | --- | --- | --- | --- | --- | --- |
|  |  | Guiyang | Anshun | Tongren | Zunyi | Qiannan |
| SEA | 12 | 2 | 4 | 2 | 1 | 3 |
| SEB | 6 | 3 | 2 | 0 | 1 | 0 |
| SEC | 1 | 0 | 0 | 1 | 0 | 0 |

SEA: enterotoxin A; SEB: enterotoxin B; SEC, enterotoxin C

**Table S5.** Virulence gene profiles of *S. aureus* food poisoning strains

| Virulence factors | Toxin protein | Virulence gene | Number of strains carrying virulence genes | Carriage rate of virulence gene (%) |
| --- | --- | --- | --- | --- |
| Superantigen exotoxins | SEA | *sea* | 12 | 63.16 |
|  | SEB | *seb* | 6 | 31.58 |
|  | SEC | *sec* | 2 | 10.53 |
|  | SEG | *seg* | 3 | 15.79 |
|  | SEH | *seh* | 1 | 5.26 |
|  | SEI | *sei* | 3 | 15.79 |
|  | SEK | *sek* | 3 | 15.79 |
|  | SELs | *selk* | 5 | 26.32 |
|  |  | *sell* | 2 | 10.53 |
|  |  | *selm* | 3 | 15.79 |
|  |  | *seln* | 3 | 15.79 |
|  |  | *selo* | 3 | 15.79 |
|  |  | *selp* | 4 | 21.05 |
|  |  | *selq* | 4 | 21.05 |
|  |  | *selu* | 2 | 10.53 |
|  | SEM | *sem* | 2 | 10.53 |
|  | SEN | *sen* | 2 | 10.53 |
|  | SEO | *seo* | 2 | 10.53 |
|  | SEP | *sep* | 2 | 10.53 |
|  | SEQ | *seq* | 4 | 21.05 |
|  | TSST-1 | *tsst-1* | 1 | 5.26 |
| Cytotoxins | PVL | *lukF-PV* | 1 | 5.26 |
|  |  | *lukS-PV* | 1 | 5.26 |
|  | α- hemolysin | *hly/hla* | 17 | 89.47 |
|  | β- hemolysin | *hlb* | 19 | 100.00 |
|  | δ- hemolysin | *hld* | 17 | 89.47 |
|  | γ- hemolysin | *hlgA* | 19 | 100.00 |
|  |  | *hlgB* | 10 | 52.63 |
|  |  | *hlgC* | 11 | 57.89 |
| Extracellular enzymes | Coa | *coa* | 16 | 84.21 |
|  | TNase | *nuc* | 19 | 100.00 |
| Adhesion-related factors | Clumping factors | *clfA* | 18 | 94.73 |
|  |  | *clfB* | 19 | 100.00 |
|  | Fibronectin-binding protein | *fnbA* | 14 | 73.68 |
|  |  | *fnbB* | 8 | 42.11 |
|  | SpA | *spa* | 19 | 100.00 |

SEA: enterotoxin A; SEB: enterotoxin B; SEC: enterotoxin C; SEG, enterotoxin G; SEH, enterotoxin H; SEI, enterotoxin I; SEK, enterotoxin K; SEM, enterotoxin M; SEN, enterotoxin N; SEO, enterotoxin O; SEP, enterotoxin P; SEQ: enterotoxin Q; TSST-1: toxic shock syndrome toxin-1; PVL: panton-valentine leucocidin; Coa: staphylocoagulase; TNase: thermostable nuclease; SpA: protein A
